# Sepsis: thrombocytopenia is bad, not recovering thrombocytopenia is too bad

**DOI:** 10.1186/cc9860

**Published:** 2011-03-11

**Authors:** W Faviere, T Boechat

**Affiliations:** 1 Universidade Severino Sombra, Vassouras, Brazil

## Introduction

Thrombocytopenia is a prognostic marker in the critically ill population [1], affecting, indistinctly, patients presenting low platelet count on admission or developing it during their stay in the ICU. It has been shown that a drop in platelet count to ≤50% of admission is associated with high death rates [2]. We aimed to observe the outcome of thrombocytopenic septic patients in our ICU.

## Methods

A retrospective observational cohort study in an 11-month period (August 2009 to July 2010) in an eight-bed medical-surgical ICU at a university hospital. This study included patients who fulfilled the criteria for sepsis as defined in the Surviving Sepsis Campaign and excluded those who spent less than 24 hours in the ICU. Thrombocytopenia (T) was defined as platelet count <150 × 10^9^/l, recovering thrombocytopenia (RT) platelet count returning to >150 × 10^9^/l and not recovering thrombocytopenia (NRT) platelet count consistently <150 × 10^9^/l. We focused on the demographic data, APACHE II score, platelet count on admission, platelet count during stay and platelet count at the time of discharge from ICU. The primary outcome was ICU mortality.

## Results

Complete data were available for 62 patients. Six were excluded. Twenty-eight males (50%), mean age 58 years (12 to 88 years), median APACHE II score 16.7 (interval 2 to 37). During the sepsis course 34 patients (60.7%) developed T, 15 (44.1%) had a drop in platelet count to <50% of admission and NRT occurred in 18 (53%). Mortality in the T group was 76.4% (RR = 1.9; 95% CI = 1.17 to 2.74; *P *< 0.01), in platelet count drop to <50% of admission group it was 93.3% (RR = 1.47; 95% CI = 1.02 to 2.2; *P *< 0.05), and in RT patients 50% survived to be discharged from the ICU. In the NRT group the mortality was 100% (RR = 2; 95% CI = 1.3 to 3; *P *< 0.001) while in nonthrombocytopenics the total mortality was 40.9% (*P *< 0.01). In T group patients the APACHE II score did not predict accurately the mortality risk. In both APACHE II groups (> 22 (*P *= 0.007) or <22 (RR = 1.7; *P *= 0.05)) thrombocytopenia was highly associated with death. The ICU overall mortality in this period was 32%.

## Conclusions

Thrombocytopenia - and its behavior - is a simple prognostic marker for ICU mortality independently of and comple-mentary to established severity of disease scores. For septic patients thrombocytopenia is bad, not recovering thrombocytopenia is worse.
